# Fine-tuning sequence-to-expression models on personal genome and transcriptome data

**DOI:** 10.1186/s13059-026-04091-1

**Published:** 2026-05-25

**Authors:** Ruchir Rastogi, Aniketh Janardhan Reddy, Ryan Chung, Nilah M. Ioannidis

**Affiliations:** 1 Department of Electrical Engineering and Computer Sciences, University of California Berkeley, Berkeley, CA USA; 2 Center for Computational Biology, University of California Berkeley, Berkeley, CA USA; 3 Department of Applied Mathematics, University of California Santa Cruz, Santa Cruz, CA USA; 4 UCSC Genomics Institute, University of California Santa Cruz, Santa Cruz, CA USA; 5 Chan Zuckerberg Biohub, San Francisco, CA USA

## Abstract

**Background:**

Genomic sequence-to-expression deep learning models, which are trained to predict gene expression and other molecular phenotypes across the reference genome, have recently been shown to have poor out-of-the-box performance in predicting gene expression variation across individuals based on their personal genome sequences.

**Results:**

Here, we explore whether additional training (fine-tuning) on paired personal genome and transcriptome data improves the performance of such sequence-to-expression models. Using Enformer as a representative pre-trained model, we explore various fine-tuning strategies. Our results show that fine-tuning improves expression predictions on held-out individuals, including from held-out populations, for genes seen during fine-tuning, with comparable performance to variant-based linear models commonly used in transcriptome-wide association studies. However, fine-tuning does not improve model generalizability to held-out genes, which contain sequences and variants unseen during fine-tuning.

**Conclusions:**

Including individual-level genetic variation and paired expression data during the training of sequence-to-expression models improves their understanding of seen variants, enabling their application to held-out individuals. However, this strategy does not improve generalizability to unseen genes, highlighting a remaining open challenge in the field.

**Supplementary Information:**

The online version contains supplementary material available at 10.1186/s13059-026-04091-1.

## Background

Predicting the effects of noncoding variants on gene expression is a key goal for understanding gene regulation and advancing personalized medicine. Observational studies of gene expression across many individuals have identified variants in expression quantitative trait loci (eQTLs) associated with differences in the expression of particular genes. These datasets have also been used to train linear models, such as PrediXcan [1] and BLUP [2], to predict gene expression from the dosages (i.e. allele counts) of common variants surrounding each gene. These models, which we collectively term variant-based models to emphasize that they ignore the sequence context surrounding a variant, are commonly used in transcriptome-wide association studies (TWAS) [1, 3].

Recently, sequence-to-expression deep learning models have been proposed to predict gene expression (as well as other molecular phenotypes related to gene regulation) from the underlying reference genome sequence across a wide variety of cell types and tissues [4–9]. Because these deep learning models use sequence rather than variant dosages as their input, they can make predictions for any sequence, including personal genome sequences containing any combination of variants. In theory, the potential advantages of sequence-based deep learning models over variant-based linear models when applied to personal genomes include the ability to (1) account for the effects of rare or *de novo* variants, which often have large effects on expression [10, 11]; (2) prioritize causal variant effects on gene expression rather than non-causal associations, informed by an understanding of regulatory sequence grammar; and (3) account for non-linear effects and interactions between variants.

Typically, sequence-to-expression models are evaluated on their ability to predict gene expression at held-out locations along the reference genome. They have also been evaluated on variant interpretation tasks such as distinguishing fine-mapped eQTLs from matched background variants [7], but such evaluations are complicated by uncertainty surrounding true causal variants in association studies, which is an ongoing challenge due to linkage disequilibrium (LD) [12]. Therefore, to directly measure performance on variants contained in personal genomes, recent studies evaluated the ability of current sequence-to-expression models to explain variation in gene expression levels across individuals [13, 14]. These studies found that current sequence-to-expression models applied to personal genome sequences are unable to explain variation in expression across individuals (cross-individual variation) for most genes. In some cases, strong negative correlations between predicted and measured expression levels suggested that models have difficulty predicting the direction of effect of regulatory variation on expression in a personal genome context. Additional work has also shown that sequence-based predictions on personal genomes are often highly uncertain [15].

Because existing sequence-to-expression models were solely trained on reference genome sequences, we hypothesized that seeing examples of genetic variation and paired expression data during training would enable them to better learn the effects of small sequence differences. To test this hypothesis, we fine-tuned an existing sequence-based model on paired personal genome and transcriptome data in lymphoblastoid cell lines (LCLs) from the Geuvadis consortium [16]. We used Enformer [7] as the pre-trained genomic deep learning model since it has been widely used to model gene expression, chromatin accessibility, histone modifications, and transcription factor binding across many cell types. We tested multiple strategies for fine-tuning Enformer using the paired personal data and compared performance relative to the baseline (non-fine-tuned) Enformer and to several variant-based models commonly used in TWAS. Our top-performing fine-tuned model matches or slightly outperforms variant-based models trained on the same individual-level data. Both the fine-tuned sequence-based and the variant-based models show largely linear, additive behavior, scale similarly with training data size, and rely primarily on common variants. Nevertheless, they rely on markedly different sets of predictive variants. Importantly, fine-tuning improves expression prediction only for genes whose individual-level data were seen during fine-tuning and does not boost generalizability to other genes, underscoring a key remaining challenge in the field.

## Results

### Model training

We fine-tuned Enformer using paired whole-genome sequencing and LCL RNA-seq data from 421 individuals in the Geuvadis consortium. For computational tractability, input sequences were restricted to $\sim$49.2 kb of personal sequence surrounding the transcription start site (TSS) of each gene—one quarter of Enformer’s original receptive field. Although this truncation omits some distal regulatory elements, linear variant-based model baselines using only variants within this window recover over 95% of their maximal predictive performance, suggesting that most *cis*-regulatory signal explaining expression differences between individuals resides in this region (Additional file 1: Fig. S1). We compare to the variant-based model baselines with this same $\sim$49.2 kb context size in all subsequent analyses.

**Fig. 1:**
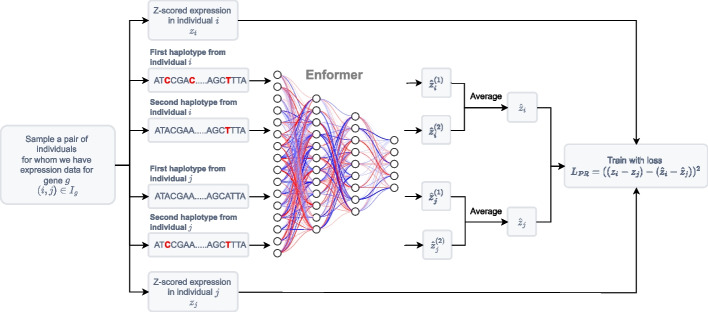
Pairwise regression training schematic. During fine-tuning, a gene *g* is sampled from a set of genes *G*. From the training individuals $I_{g}$ for gene *g*, a pair of individuals (*i*, *j*) is randomly sampled, along with their *z*-scored expression levels $z_i$ and $z_j$. Enformer is fine-tuned to predict the expression difference $z_i - z_j$ from the individuals’ personal genome sequences using a mean squared error loss between the actual difference and the predicted difference $\hat{z}_i - \hat{z}_j$. Red nucleotides highlight genetic variants in each haplotype

We evaluated multiple strategies for fine-tuning Enformer. For the main results shown below, rather than fine-tuning on personal sequences one at a time (an approach we refer to as single-sample regression), we adopted a contrastive loss that compares the predicted expression difference between two individuals for a given gene to the true difference. This loss encourages models to attribute differences in expression between two individuals to the small number of sequence variants between them. We implemented two versions of these pairwise models: one with a regression loss and another with a classification loss. Since both pairwise models have comparable performance, we focus on the pairwise regression model throughout unless otherwise specified. Figure 1 shows a schematic of the pairwise regression model, and Additional file 1: Fig. S2 also illustrates the single-sample regression and pairwise classification models.

### Fine-tuning splits and evaluation setup

**Fig. 2:**
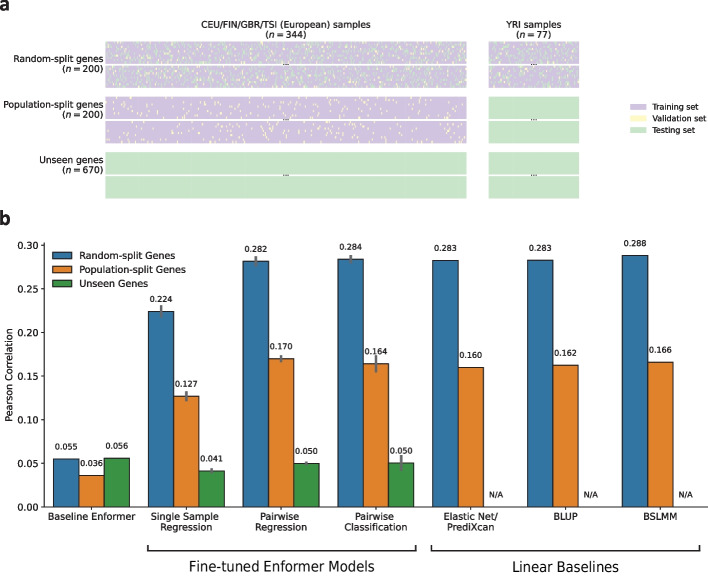
Evaluation setup and model performance. **a** The three gene sets used for model evaluation: random-split genes, population-split genes, and unseen genes. Each row represents a gene and each column an individual, with colors indicating membership in the training, validation, or testing sets. 20 example genes are shown for each gene set. Individuals in the dataset come from five populations: CEU (Utah residents with Northern and Western European ancestry), FIN (Finnish in Finland), GBR (British in England and Scotland), TSI (Toscani in Italy), and YRI (Yoruba in Ibadan, Nigeria). **b** Performance of fine-tuned Enformer models compared to baselines in predicting gene expression across individuals. We trained three replicates for each of the fine-tuned Enformer models using different random seeds, which shuffled the data order and pairs sampled (for pairwise models) while keeping the set of training individuals per gene fixed. Bar heights indicate the mean (over genes) of the cross-individual Pearson correlations between measured and predicted expression levels. For fine-tuned models, bar heights are averaged across three replicates, with error bars showing the standard deviation

To assess model performance under varying levels of generalization difficulty, we constructed three groups of genes with different partitions of individuals into training, validation, and testing sets (Fig. 2a). The first group (random-split genes; $n = 200$) evaluates generalization to randomly held-out individuals, mirroring the standard evaluation of variant-based models used in TWAS. The second group (population-split genes; $n = 200$)—in which models are trained on individuals of European ancestry and tested on individuals of Yoruba ancestry—gauges cross-population generalizability, a known limitation of variant-based models [17, 18]. The third group (unseen genes; $n = 670$) assesses generalization to genes absent during fine-tuning, testing whether fine-tuning on some genes improves the learned *cis*-regulatory grammar in a way that is generalizable to sequence variation around other genes. These non-overlapping groups of genes were randomly selected from the 3,259 genes with at least one significant eQTL in Europeans in the original Geuvadis analysis.

All fine-tuned models were trained jointly on the 400 random-split and population-split genes. To ensure fair comparison, all models (including the variant-based models used for benchmarking) were trained on identical sets of individuals. Training and inference costs for both the fine-tuned Enformer models and variant-based linear baselines are included in Additional file 1: Table S1.

### Performance on random-split genes

For random-split genes, where individuals are randomly assigned to training and test sets regardless of ancestry, test set individuals largely share alleles—especially common ones—with training set individuals (Additional file 1: Fig. S3). Thus, this evaluation primarily measures how well models generalize to sequences containing new combinations of already-seen variants and haplotypes, with relatively few rare or private variants found only in test set individuals.

For each gene, we measure performance by computing the Pearson correlation across individuals in the test set. Fine-tuned models that use a pairwise loss, either regression or classification, have a mean correlation ($r = 0.28$) that substantially outperforms baseline Enformer ($r = 0.06$) and matches variant-based linear models (Fig. 2b, Additional file 1: Fig. S4, and Additional file 1: Fig. S5a), which have been shown to approach narrow-sense SNP heritability in this setting [1, 3]. The fine-tuned model that uses a single-sample loss ($r = 0.23$) performs worse than either pairwise model, illustrating the utility of the pairwise loss for learning accurate variant effect sizes.

### Performance on population-split genes

Variant-based models typically perform substantially worse when applied to individuals from populations they were not trained on for two key reasons: differences in linkage disequilibrium (LD) patterns between populations, and the presence of causal variants in test populations that are rare or absent in training populations [19]. Indeed, many alleles—including common ones—appear only in the Yoruba individuals in our dataset (Additional file 1: Fig. S3). Fine-tuned sequence-based models might handle these challenges better than variant-based models, particularly if their pre-training on genome-wide functional data provides an understanding of sequence grammar that helps them pinpoint causal variants within LD blocks.

However, we find that fine-tuned sequence-based models have similarly degraded performance on population-split genes, where Yoruba individuals are held out during fine-tuning, relative to their performance on random-split genes ($r = 0.17$ vs. 0.28 for pairwise regression, Fig. 2b). To assess whether this decline is driven by alleles private to Yoruba individuals not seen during fine-tuning, we computed, across genes, the correlation between the proportion of unseen alleles and the proportion of narrow-sense SNP heritability explained by the pairwise regression model (Additional file 1: Fig. S6). The observed correlation is negative, as expected, but weak ($r=-0.14$), indicating that unseen alleles only partially account for the performance drop. We also do not find evidence of fine-tuned models overfitting by assigning zero effects to unseen alleles (Additional file 1: Fig. S7). These findings point to poor causal variant identification as the principal cause of reduced performance on population-split genes. Still, the top-performing pairwise regression model slightly outperforms the best variant-based model baseline for population-split genes (Fig. 2b and Additional file 1: Fig. S5b), which was not the case for random-split genes, indicating that fine-tuned sequence-based models might have a slight advantage in identifying causal variants, a point we examine in more detail below.

### Performance on unseen genes

Model performance on unseen genes reflects the ability to generalize to sequences containing variation entirely absent from the training data, similar to the setting in which baseline Enformer and other sequence-to-expression models were originally shown to have poor performance. However, learning from examples of personal sequence variation during fine-tuning on other genes might improve upon baseline Enformer’s understanding of *cis*-regulatory grammar in a way that is generalizable to unseen variation in unseen genes. Performance on this task is also important because a model capable of generalizing to unseen genes should similarly generalize to unseen variants in seen genes, including rare and *de novo* variants of clinical importance. Note that this evaluation focuses exclusively on the fine-tuned models and baseline Enformer, since variant-based models cannot be applied to unseen genes or variants, as they infer effects only for variants in their training data.

Despite their much improved performance on the 400 random-split and population-split genes shown above, fine-tuned models perform comparably to or slightly worse than baseline Enformer on unseen genes, regardless of the specific fine-tuning loss used (Fig. 2b). This pattern persists even when considering absolute values of cross-individual correlations (Additional file 1: Fig. S8). In addition, fine-tuned model performance on individual unseen genes varies substantially (Additional file 1: Fig. S9), with numerous genes having negative correlations, as was previously observed for baseline Enformer [13, 14]. Comparing performance between the pairwise regression and baseline Enformer models on individual unseen genes (Additional file 1: Fig. S9) reveals greater consistency in correlation magnitudes than in directions, echoing previous findings comparing different sequence-to-expression models [13] or replicates of the same model class [15]. These results suggest that fine-tuning on individual-level data alone is insufficient for learning an improved, generalizable representation of *cis*-regulatory grammar.

### Ablation experiments

To better understand the mechanisms underlying our results and explore strategies for improvement, we additionally conducted a series of ablation experiments.

**Fig. 3:**
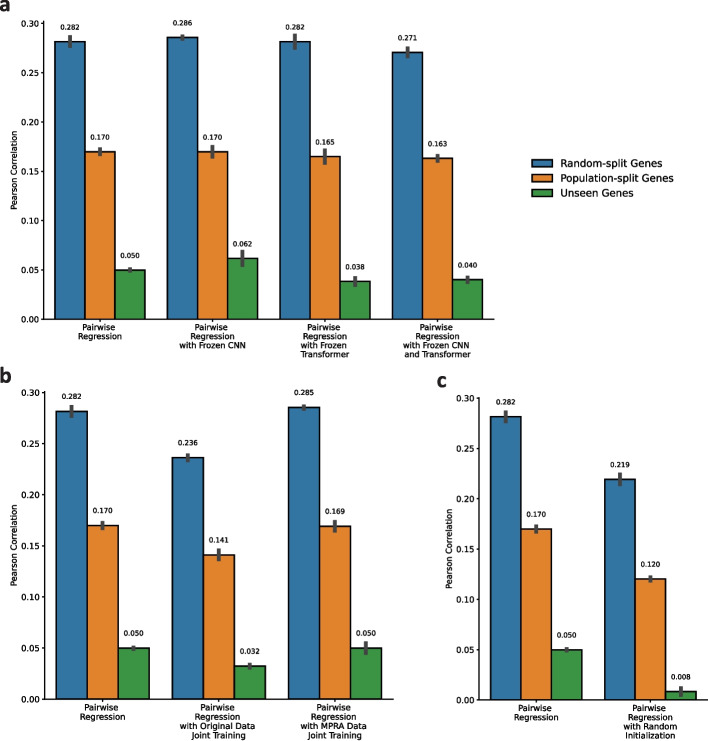
Ablation analysis. Performance of the pairwise regression model on the three evaluation sets when **a** freezing model components during fine-tuning; **b** fine-tuning on individual-level data together with either Enformer’s original training data or MPRA data from Siraj et al. [20]; and **c** starting from randomly initialized weights. Bar heights indicate mean Pearson correlations across genes and three replicates, and the error bars indicate the standard deviation of the replicate means

First, we froze various components of the Enformer architecture during fine-tuning to determine which parts were responsible for the improved performance. Enformer consists of a convolutional neural network (CNN) that captures local sequence information, transformer layers that share information across genomic bins, and a two-layer multi-layer perceptron (MLP) that maps embeddings to predictions. Freezing either the CNN layers, the transformer layers, or both the CNN and transformer layers only minimally degrades performance (Fig. 3a), suggesting that fine-tuning predominantly adjusts the final two-layer MLP connecting transformer outputs to predictions. This limited scope of change may explain the lack of generalization to unseen genes, as the core *cis*-regulatory grammar learned by the fine-tuned models is largely unchanged.

We also explored whether augmenting the personalized expression data with additional data during fine-tuning could boost performance. First, we investigated potential catastrophic forgetting, a phenomenon in which fine-tuned models overfit the new data and lose knowledge from their original training tasks necessary for generalization [21, 22]. While we observed that the fine-tuned models perform only slightly worse than baseline Enformer on Enformer’s original human tasks (Additional file 1: Fig. S10), to entirely avoid any forgetting [23, 24] we also tried fine-tuning jointly on both the personal data and Enformer’s original training data. However, this approach did not improve performance (Fig. 3b). We also attempted to expose the model to extra sequence variation during fine-tuning by supplying data from more than 200,000 trait-associated variants whose effects were measured in Massively Parallel Reporter Assay (MPRA) experiments in five cell types, including a lymphoblastoid cell line [20]. While MPRA sequences are much shorter (200 bp) than the personal genome sequences used for fine-tuning and are not assayed in an endogenous genomic context, we hypothesized that this additional sequence variation data might provide a complementary training signal. However, joint fine-tuning with the MPRA data also did not improve performance (Fig. 3b).

Finally, to assess the importance of pre-training for the fine-tuning task, we also trained a pairwise regression model with the same architecture but initialized with random weights rather than the pre-trained Enformer weights. This randomly initialized model performed markedly worse (Fig. 3c), highlighting the importance of pre-training on genome-wide functional assays for stable and effective fine-tuning, given the limited size of the individual-level training data.

### Prioritization of putatively causal variants

Models with the same cross-individual performance might still attribute regulatory effects to different variants. Therefore, beyond evaluating predictive performance, we also probed the internal behavior of our fine-tuned models by assessing whether the variants that most influence model predictions in seen genes are enriched for putatively causal regulatory variants, using two complementary analyses.

**Fig. 4:**
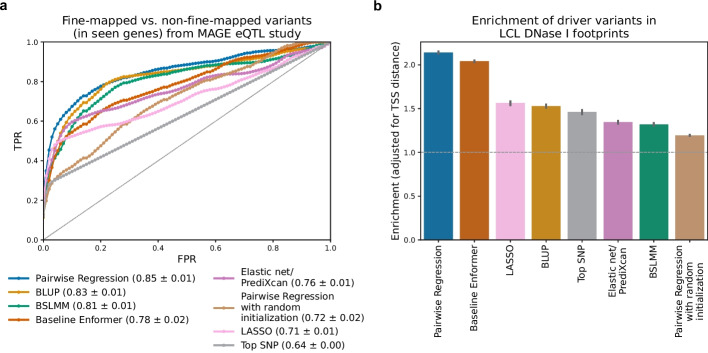
Causal variant prioritization. **a** ROC curves of model performance at prioritizing fine-mapped eQTLs (PIP > 0.9) over non-fine-mapped eQTLs (PIP < 0.01) from the same genes, based on fine-mapping results from the MAGE LCL eQTL study. Analysis was restricted to eQTLs encountered by all models during training. AUROC means and standard deviations were computed across 100 randomly subsampled datasets, where for each fine-mapped eQTL, one non-fine-mapped eQTL from the same gene was randomly selected. **b** Enrichment of model drivers (from all 400 random-split and population-split genes) overlapping LCL DNase I footprints, adjusted for the TSS distance of drivers. Bar heights show mean enrichments across 1000 bootstraps in which drivers were sampled with replacement; error bars indicate the 95% confidence interval of the mean

First, we utilized fine-mapping results for LCL eQTLs from the large and globally diverse MAGE study [25]. For each model, we compared the effect sizes assigned to likely causal eQTLs (PIP > 0.9) to those assigned to likely non-causal eQTLs (PIP < 0.01) from the same genes. The fine-tuned pairwise regression model most accurately distinguishes between these two classes of variants (Fig. 4a). Notably, baseline Enformer also outperforms several variant-based models on this task, despite not having seen the variants during training, suggesting that its poor cross-individual predictions are the result of miscalibrated effect sizes or sign errors rather than an inability to identify causal variants. In contrast, the randomly-initialized pairwise model underperforms nearly all other baselines, underscoring the importance of pre-training.

Next, we identified each model’s driver variants [14]—those with the largest influence on predictions—for the genes seen during fine-tuning and measured their overlap with sites of transcription factor occupancy identified by DNase I footprinting in LCLs [26]. Overlapping variants might causally affect gene expression by disrupting transcription factor binding. A raw overlap comparison would be confounded by non-uniform footprint density around TSSs (Additional file 1: Fig. S11) and by model architecture biases that affect where drivers are identified relative to TSSs (Additional file 1: Fig. S12). For instance, variant-based linear models, which do not take position as input, have a roughly uniform distribution of drivers across distances. We therefore computed an enrichment conditional on TSS distance, quantifying whether each model’s drivers overlap footprints more than expected given their TSS distances (Fig. 4b). Again, the fine-tuned pairwise regression model has the highest enrichment, followed closely by baseline Enformer. All variant-based models, along with the randomly-initialized pairwise regression model, have significantly lower enrichment.

Together, these results indicate that fine-tuned models leverage the regulatory grammar learned during pre-training to more effectively prioritize causal variants during fine-tuning.

### Linear approximation of fine-tuned models

**Fig. 5:**
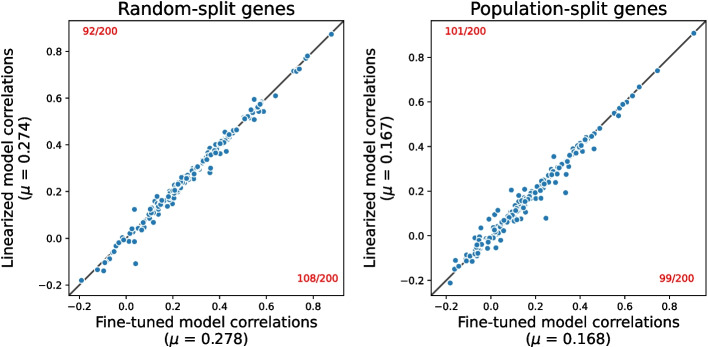
Performance comparison of fine-tuned Enformer to a linear approximation. For random-split genes (left) and population-split genes (right), we compare the cross-individual Pearson correlations of the pairwise regression model (*x*-axis) and a linear approximation (*y*-axis), termed the linearized model. Each point represents a distinct gene. The numbers of genes lying above and below the black $y = x$ line are indicated in red. The linearized model is a weighted linear combination of variant dosages, where the weights are ISM scores

The similar performance of the fine-tuned models and the variant-based linear models on held-out individuals suggests that the fine-tuned models primarily learn linear combinations of variant effects, rather than strong non-linear effects or interactions between variants. Indeed, when we construct a linear approximation of the fine-tuned pairwise regression model using its in silico mutagenesis (ISM) scores as the weights for variant dosages in a linear model, we find very similar performance between the original fine-tuned model and its linear approximation (Fig. 5). This analysis indicates that although deep learning models have the capacity to learn complex interactions, their predictions of variant effects from personal genomes are primarily linear without strong interactions between variants. Note that this does not imply that genomic deep learning models fail to learn any interactions between positions within an input sequence, such as interactions between neighboring bases within transcription factor binding motifs. Rather, it suggests that the learned variant effects at their typical spacing in personal genomes ($\sim$1kb) are approximately linear and additive.

### Contribution of rare variants to fine-tuned model performance

**Fig. 6:**
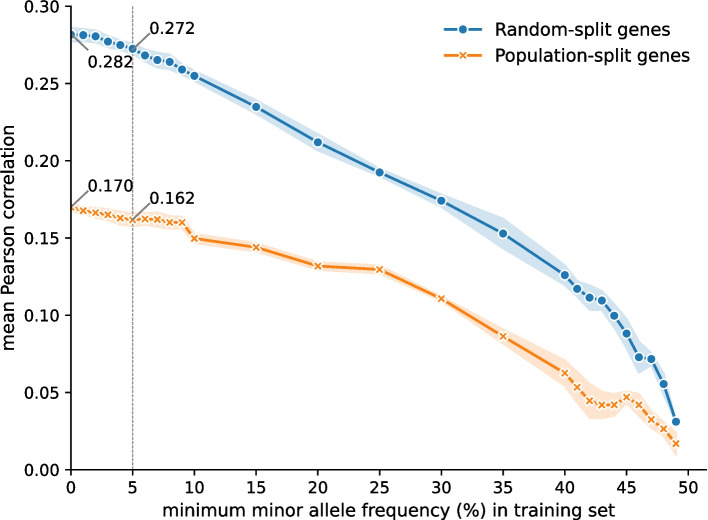
Contribution of rare variants to fine-tuned Enformer performance. To assess the impact of variant frequency on model predictions, we replaced alleles below specified minor allele frequency (MAF) thresholds (*x*-axis) with their corresponding major alleles in personal sequences. These modified sequences were then input into the pairwise regression model to generate predictions. The *y*-axis shows the mean cross-individual Pearson correlation averaged across genes and three replicates, with shaded regions indicating the standard deviation across replicates. Performance numbers are displayed for the cases when all alleles or only common alleles (MAF $\ge 5\%$) are included

Unlike variant-based linear models, which exclude rare variants as potential features due to sample size limitations, sequence-based models can potentially learn their effects. To assess whether the fine-tuned sequence-based models actually rely on rare variants, we evaluated the fine-tuned pairwise regression model on partial personal genome sequences. In these sequences, minor alleles were retained only if their frequency among training individuals exceeded a given threshold; otherwise, they were replaced with their corresponding major allele. By varying this threshold (Fig. 6), we find that common variants contribute the most to cross-individual performance, though rare variants (minor allele frequency $< 5\%$ in training set) still provide a small additional contribution.

### Performance scaling with training cohort size

**Fig. 7:**
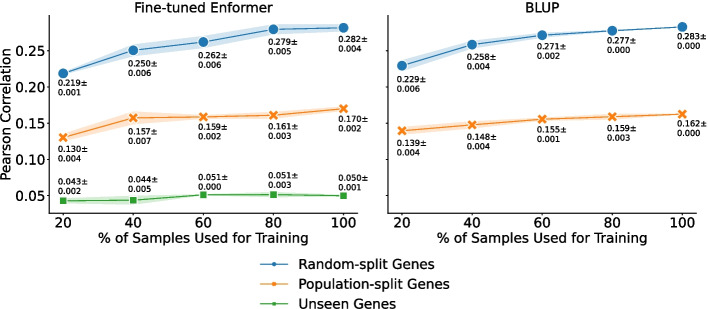
Performance scales with training set size. We compare the performance of the fine-tuned pairwise regression model (left) and BLUP (right) as a function of the fraction of training individuals used. Mean cross-individual Pearson correlations and standard deviations are calculated across three replicates, each trained on a different subsampled set of individuals. As BLUP cannot make predictions for unseen genes, performance on unseen genes is excluded from the BLUP panel

Lastly, we examined how performance scales with the number of individuals used to train models. To do so, we retrained models using randomly subsampled fractions of the available training sequences in random-split and population-split genes and then evaluated on the same held-out test individuals as in previous analyses. For seen genes, both the fine-tuned sequence-based model and the variant-based linear models show similar scaling behavior: performance improves comparably as more personal sequences are used in training (Fig. 7). In contrast, for unseen genes, the fine-tuned model’s performance does not correlate with the number of personal sequences observed in seen genes.

## Discussion

In this study, we find that multiple strategies for fine-tuning Enformer on personal genomic sequences and matched transcriptome data improve its ability to predict inter-individual expression differences in held-out individuals, a task with which the original Enformer model struggles. In agreement with recent results from a similar fine-tuning study [27], we find that this improved performance is comparable to that of variant-based linear models, which explicitly use variant dosages as predictors. Both approaches rely primarily on linear combinations of common variant effects for their predictions. Both approaches also have similar scaling with the number of individuals seen during training, but with diminishing returns as more individuals are added, likely due to the large amount of shared variation between individuals in human populations. Alternative approaches to increase the amount of sequence diversity seen during training may be helpful, although fine-tuning jointly with MPRA variant effect data did not improve performance in our analyses.

Despite their similar overall performance, fine-tuned sequence-based models and variant-based linear models differ in which variants around a gene most strongly influence model predictions. Variants with large effects in the fine-tuned models are more likely to be located within transcription factor occupancy sites and also more likely to have been fine-mapped as likely causal eQTLs. The same pattern does not hold for sequence-based models initialized with random weights rather than pre-trained Enformer weights, suggesting that functional sequence patterns learned by the pre-trained Enformer model enable the fine-tuned models to better prioritize variants that fall within important regulatory elements or motifs.

Importantly, although fine-tuning on personal data enables the sequence-based models to learn observed associations between variants and gene expression levels, the fine-tuned models do not generalize to new sequences containing new variants, as reflected in their poor performance on unseen genes. Developing sequence-to-expression models with an understanding of regulatory variation that is fully generalizable to unseen sequences and variants will likely require new types of data or new strategies for utilizing observed variation during training, an important area for future work in the field.

## Conclusions

We explored whether fine-tuning sequence-to-expression deep learning models using paired personal genome and transcriptome data improves their ability to predict cross-individual variation in gene expression, a key personal genome interpretation task with which state-of-the-art models struggle. We found that fine-tuning improves expression predictions on held-out individuals for genes seen during fine-tuning, with comparable performance to variant-based linear models both within and across populations. However, we found that fine-tuning does not improve model generalizability to held-out genes, which contain sequences and variants unseen during fine-tuning, highlighting a remaining open challenge in the field.

## Methods

### Gene expression dataset

We trained and evaluated models using RNA sequencing (RNA-seq) data from lymphoblastoid cell lines (LCLs) provided by the Geuvadis consortium [16]. This dataset includes 421 individuals with corresponding phased whole-genome sequencing (WGS) data in the 1000 Genomes Project [28]. These 421 samples come from five populations: 92 Tuscan (TSI), 89 Finnish (FIN), 85 British (GBR), 78 European individuals from Utah (CEU), and 77 Yoruba (YRI).

To prepare the RNA-seq data, we preprocessed the uncorrected RPKM data from the GD660.GeneQuantRPKM.txt.gz file. First, to facilitate better comparisons of RNA abundances across samples, we converted gene expression measurements from RPKM to TPM using the formula

$$\begin{aligned} \text {TPM}_{g}^{(i)} = \frac{\text {RPKM}_{g}^{(i)}}{\sum \nolimits _{g'}\text {RPKM}_{g'}^{(i)}} \cdot 10^{6} \end{aligned}$$

where *g* denotes a specific gene and *i* denotes a specific individual. We then excluded poorly-expressed genes with zero read counts in at least 50% of the samples. To normalize the expression distribution of each gene, we applied a log transformation to the TPM values, using a pseudocount of 0.01. Finally, to correct for potential batch effects and account for population structure, we regressed out the first 10 gene expression principal components [29]. We denote these standardized log TPM values by *y*. At times, to equalize cross-individual variances across genes, we further *z*-score *y*, yielding values denoted by *z*.

### Data splits

To assess model performance, we constructed three disjoint datasets of increasing difficulty. From the 3,259 genes with a statistically significant eQTL (FDR < 0.05) in the Geuvadis EUR *cis*-eQTL analysis, we randomly selected (1) 200 genes for random-split evaluation, designed to test model performance on held-out individuals; (2) 200 genes for population-split evaluation, intended to assess generalization across populations; and (3) 670 genes for unseen-gene evaluation, aimed at evaluating model performance on entirely new genes and variants. To prevent data leakage, we ensured that genes selected for each evaluation set were located on different chromosomes.

For random-split genes, we randomly partitioned the 421 individuals into a development set of 344 samples and a testing set of 77 samples, irrespective of their population. The development set was further divided into a training set of 327 individuals and a validation set of 17 individuals. For population-split genes, all 344 individuals from the European super-population (comprising TSI, FIN, GBR, and CEU populations) formed the development set, which was further randomly divided into training and validation sets of the same size as before. The testing set consisted of the 77 Yoruba (YRI) individuals. For unseen genes, all 421 individuals were in the testing set.

### Fine-tuning

We fine-tuned Enformer [7] on personalized genome sequences of Geuvadis individuals. As in Huang et al. [13], personalized genome sequences for each individual’s haplotypes were generated using bcftools consensus [30] with hg19 as the reference genome, consistent with the Geuvadis variant calls. Only single-nucleotide variants were incorporated into these sequences. For computational tractability, we shortened personal genome sequences to span the 49,152 base pairs centered around the transcription start site (TSS) of each gene, as annotated in Gencode v12—which served as the same reference for RNA-seq quantification. This sequence length corresponds to approximately one-fourth of Enformer’s receptive field.

We fine-tuned the PyTorch implementation of Enformer available in the enformer-pytorch repository (version 0.8.8) [31]. For all non-ablation analyses, we fine-tuned the entire Enformer model—without freezing any weights—using the various approaches detailed below. All models were trained jointly on the 400 random-split and population-split genes. During training, we augmented the dataset by randomly reverse-complementing input sequences and shifting them by up to three base pairs in either direction (padding with zeros as necessary). We used the AdamW optimizer [32] with a learning rate that linearly increased to its peak over 1,000 steps and then decayed to zero using cosine annealing over 50 epochs. Experiments were conducted using eight GPUs (NVIDIA A5000s or TITAN RTXs) with a batch size of eight for most models and sixteen for the single-sample regression model. Two hyperparameter configurations were evaluated: (1) a learning rate of $5 \times 10^{-4}$ and weight decay of $5 \times 10^{-3}$, and (2) a learning rate of $1 \times 10^{-4}$ and weight decay of $1 \times 10^{-3}$. The configuration yielding the lower validation loss was selected. To prevent overfitting, we applied early stopping with a patience of five epochs based on the validation loss. During inference, individual-level predictions were obtained by averaging the four predictions from the forward and reverse strand sequences of both haplotypes.

We now describe the specific model architectures and loss functions used in our various approaches.

#### Single-sample regression

The single-sample regression model directly predicts a single individual’s gene expression from their two haplotypes (Additional file 1: Fig. S2a). Because Enformer does not natively have an LCL RNA-seq output track, we introduced an additional output head to generate predictions. Briefly, Enformer generates embeddings of size $384 \times 3072$ for each haplotype. (There are 384 bins—each of size 128 bp—in the truncated 49,152 bp sequence, and 3072 is the embedding dimensionality.) The central ten bins of the embedding were aggregated using attention pooling, which performs a learned weighted linear combination. Predictions were then made by applying a linear layer to the pooled embeddings. The final gene expression prediction was obtained by averaging the predictions from both haplotypes.

Because a naive mean-squared error (MSE) loss would bias the model towards genes with high variance, we employed the symmetric mean absolute percentage error (SMAPE) loss to quantify the relative error between the predicted value $\hat{y}$ and the true value *y*:

$$\begin{aligned} L_\text {SSR}(y, \hat{y}) = \frac{|y - \hat{y}|}{(|y| + |\hat{y}|)/2}. \end{aligned}$$

We found that a learning rate of $1 \times 10^{-4}$ and weight decay of $1 \times 10^{-3}$ was the optimal hyperparameter configuration.

#### Pairwise regression

To emphasize genomic positions where individuals differ, we adopted a pairwise regression strategy inspired by contrastive learning (Additional file 1: Fig. S2b). In this approach, we input the haplotypes of two individuals into the model. The model shares the same architecture as the single-sample regression model to generate predictions for each individual. However, instead of penalizing individual prediction errors, the model was trained to predict the expression difference between two individuals using the following pairwise loss:

$$\begin{aligned}L_{\mathrm{PR}}(z_1,z_2,{\widehat z}_1,{\widehat z}_2)=\underbrace{((z_2-z_1)}_{\substack{\mathrm{actual}\\\mathrm{difference}}}-\underbrace{({\widehat z}_2-{\widehat z}_1))}_{\substack{\mathrm{predicted}\\ \mathrm{difference}}}\end{aligned}^2$$

We utilized *z*-scored expression values to standardize gene variances, ensuring that all genes contribute equally to the loss. We found that a learning rate of $1 \times 10^{-4}$ and weight decay of $1 \times 10^{-3}$ was the optimal hyperparameter configuration.

#### Pairwise classification

In previous approaches, we introduced a new output head to make RNA-seq predictions. However, adding parameters could enable the model to adapt by only updating new components without updating the core parameters essential to learn a generalizable *cis*-regulatory grammar. Therefore, in this approach, we leveraged the model’s existing predictions for the CAGE track of a corresponding lymphoblastoid cell line, GM12878.

Because the CAGE outputs differ significantly in scale from the RNA-seq data used for fine-tuning, we reformulated the task as a binary classification problem (Additional file 1: Fig. S2c). Specifically, we trained the model to predict whether the gene expression of one individual exceeds that of another. First, we summed the ten central bins of the GM12878 CAGE prediction track for each haplotype to obtain a single expression prediction per individual. The predictions for individuals 1 and 2, $\hat{y}_{1}$ and $\hat{y}_{2}$, follow Poisson distributions. Computing $P(\hat{y}_{1}> \hat{y}_{2})$ requires the cumulative distribution function of a Skellum random variable, which is intractable. To address this, we applied the Anscombe transform to approximate the Poisson distributions as normal distributions, making the above probability computation tractable. From this, we calculated the binary cross-entropy loss between the predicted probability and the ground truth:

$$\begin{aligned} L_{\text {PC}}(y_{1}, y_{2}, \hat{y}_{1}, \hat{y}_{2}) = \text {CrossEntropy}(P(\hat{y}_{1}> \hat{y}_{2}), 1\{y_1> y_{2}\}). \end{aligned}$$

To reduce the effects of measurement noise, we applied the classification loss only to pairs whose expression percentiles for the given gene differed by at least 25%. We found that a learning rate of $1 \times 10^{-4}$ and weight decay of $1 \times 10^{-3}$ was the optimal hyperparameter configuration.

### Ablation analyses

#### Freezing model components during fine-tuning

We fine-tuned models while freezing (i.e. not updating) the CNN layers, transformer layers, or both—while allowing the remaining components to be updated. All models were trained using a learning rate of $1 \times 10^{-4}$ and weight decay of $1 \times 10^{-3}$. Freezing the CNN reduced GPU memory usage, allowing us to use a batch size of 32 when just the CNN or both the CNN and transformer were frozen. When only the transformer was frozen, we used a batch size of 8.

#### Joint training with Enformer’s original dataset

We jointly trained Enformer on both the personalized expression dataset and its original training dataset. Enformer’s original dataset consists of 196,608 base-pair sequences from human and mouse reference genomes, along with associated gene expression, chromatin accessibility, histone modification, and transcription factor binding measurements (5,313 human and 1,643 mouse). We utilized the same sequences from the original Enformer dataset as were used by Avsec et al. [7] for training. During joint training, we rotated between batches from the original human dataset, original mouse dataset, and the personalized expression dataset and applied gradient updates only after seeing one batch from each data source. We employed Enformer’s standard architecture and Poisson loss for the original data, while applying either our pairwise regression or pairwise classification architectures and loss functions to the personalized expression data. We found that a learning rate of $5 \times 10^{-4}$ and weight decay of $5 \times 10^{-3}$ was the optimal hyperparameter configuration.

#### Joint training with MPRA data

We jointly trained Enformer on both the personalized expression dataset and Massively Parallel Reporter Assay (MPRA) data. We utilized the MPRA dataset from Siraj et al. [20], which quantifies the effects of over 200,000 trait-associated variants on promoter-driven expression in five cell types: GM12878, HepG2, K562, A549, and SK-N-SH (see Additional file 1: Table S2 for ENCODE [33–35] accession IDs).

We fine-tuned on the MPRA data using a pairwise regression approach, analogous to our approach previously described for personalized expression data. Briefly, for each variant, both the 200 bp reference and alternate sequences were input into the model. Cell-type-specific expression predictions were generated for each sequence using cell-type-specific linear output heads that were applied to the flattened Enformer embeddings. The predicted cell-type-specific variant effect was calculated as the difference between alternate and reference expression predictions. This prediction was compared to the *z*-scored ground-truth effect size from the MPRA dataset using the loss function

$$\begin{aligned} L_{\text {MPRA}} = \frac{1}{5} \sum \limits _{i = 1}^{5} \left[ \underbrace{z_{c_{i}}}_{\begin{array}{c} \text {measured} \\ \text {effect} \end{array}} - \underbrace{(\hat{z}_{c_{i}}^{\text {ALT}} - \hat{z}_{c_{i}}^{\text {REF}})}_{\begin{array}{c} \text {predicted} \\ \text {effect} \end{array}}\right] ^{2}, \end{aligned}$$

where $c_{i}$ denotes the *i*-th cell type. If a variant effect was unavailable for a particular cell type, the corresponding loss term was excluded. During joint fine-tuning, we alternated between batches from the MPRA dataset and the personalized expression dataset and applied gradient updates only after seeing one batch of each. We found that a learning rate of $1 \times 10^{-4}$ and weight decay of $1 \times 10^{-3}$ was the optimal hyperparameter configuration.

#### Training randomly-initialized Enformer models on personalized expression data

We trained pairwise regression models, initialized with random weights instead of Enformer’s pre-trained weights, using only the personalized expression dataset. Compared to fine-tuning a pre-trained model, these models required a substantially lower learning rate and weight decay to achieve convergence, as well as significantly more training epochs. We used a learning rate of $1 \times 10^{-5}$ and weight decay of $1 \times 10^{-4}$. Models were trained for up to 50 epochs without early stopping, unlike their counterparts initialized with pre-trained weights that used early stopping.

### Baselines

#### Baseline Enformer

We generated predictions using the original Enformer model on the same personal genome sequences that were used for fine-tuning, which included only SNPs within the approximately 49.2 kb region centered at the gene’s TSS. For each input sequence, we obtained a prediction by averaging the central 10 output bins of the GM12878 CAGE track. Individual-level predictions were then calculated by averaging the four predictions from the forward and reverse strand sequences of both haplotypes.

#### Variant-based linear methods

We also benchmarked five variant-based linear methods commonly used in TWAS: top SNP, LASSO [36], elastic net (also known as PrediXcan) [1, 37], BLUP [2], and BSLMM [38]. These methods, implemented using the FUSION package [3], produce a separate model for each gene. Training and testing were conducted on both random-split and population-split genes, using the same sample splits as in fine-tuning. All models were trained to predict *z*-scored standardized log TPM values from SNP dosages scaled to have zero mean and unit variance. Only common SNPs (MAF $\ge$ 5% in training set samples) within the $\sim$49.2 kb context window centered at the gene’s TSS were included as predictors. The optimal hyperparameter $\lambda$ in the elastic net model was chosen through 5-fold cross-validation on the training set, consistent with FUSION’s default settings. The other methods do not have tunable hyperparameters.

### Heritability estimation

We estimated each gene’s narrow-sense SNP heritability ($h_{\text {SNP}}^{2}$) using FUSION, which calls GCTA to fit a variance-component model via restricted maximum likelihood. Models were fit using *z*-scored standardized log TPM expression values and common variants across all 421 Geuvadis individuals in our dataset. To compare model performance across genes with varying genetic architectures, we report the normalized performance metric $r_{\text {test}} / \sqrt{h^{2}_{\text {SNP}}}$, where $r_{\text {test}}$ is the cross-individual Pearson correlation on the test set.

### Computing in silico mutagenesis (ISM) scores

For the Enformer models, we calculated ISM scores for all variants in our dataset. The ISM score for each variant is determined by the formula $\text {ISM} = f(x_{\text {ALT}}) - f(x_{\text {REF}})$, where *f* predicts gene expression in LCLs. Here, $x_{\text {REF}}$ represents the reference hg19 sequence centered at the gene’s TSS, and $x_{\text {ALT}}$ is the same sequence with the variant’s alternate allele substituted at the appropriate position. We compute ISM scores separately for the forward and reverse strand sequences and then average these two values to obtain the overall ISM score. Similar to coefficients ($\beta$’s) in a linear model, ISM scores reflect the marginal change in gene expression when the dosage of the variant increases by 1.

### Identifying model drivers

To identify significant genetic variants (drivers) that influence model predictions for each gene, we employed an algorithm that accounts for correlations between variants, first proposed in Sasse et al. [14]. We start by approximating the model’s potentially non-linear prediction function with a simpler linear function. For variant-based linear models, this approximation is exact; for Enformer models, we use a linear approximation, whose accuracy was validated in Fig. 5.

Then, we rank all variants based on the magnitude of their effect sizes—coefficients for linear models and ISM scores for Enformer models. Starting with the most significant variants, we iteratively add a variant to the driver set if it meets two criteria. First, including the variant must increase the Pearson correlation between our linear approximation and the original model’s predictions by at least 0.05. Second, the variant’s individual contribution must have a significant (Bonferroni-corrected *p*-value < 0.01) and positive correlation with the model’s predictions.

### Prioritizing putatively causal variants

#### Distinguishing fine-mapped from non-fine-mapped MAGE eQTLs

We evaluated each model’s ability to distinguish likely causal variants (PIP > 0.9 according to SuSiE) from likely non-causal variants (PIP < 0.01) in seen genes using fine-mapping results from the MAGE eQTL study [25]. We chose MAGE over Geuvadis for this evaluation because MAGE provides a larger sample size (731 versus 421 individuals) and greater genomic diversity. Since MAGE reports variants in hg38 coordinates while our study was conducted in hg19, we used Picard LiftoverVcf [39] to map variant positions. We restricted our analysis to eQTLs seen by all models during training. That is, the eQTL must affect a random-split or population-split gene, be located within the $\sim$49.2 kb context window surrounding the gene’s TSS, and have MAF $\ge$ 0.05 among training set individuals in our dataset.

We assessed model performance by setting up a classification task. For each likely causal eQTL, we randomly selected a likely non-causal eQTL from the same gene, yielding a balanced dataset. We then computed the AUROC for distinguishing between these two classes based on the percentiles of predicted effect magnitudes, where percentiles were calculated relative to the set of variants seen by all models in that gene. We repeated this procedure 100 times, sampling with replacement likely non-causal eQTLs, to compute the mean and standard deviation of the AUROC.

#### Overlap of model drivers with DNase I footprints

For each model, we assessed whether its driver variants identified in random-split and population-split genes overlap DNase I footprints. We used 1% FPR footprint calls from Vierstra et al. [26], which includes data from two LCL samples: GM06990-DS7748 and GM12865-DS12436. The union of these calls was used as the footprint set for this analysis. Since our study was conducted in hg19 while the footprint calls are in hg38, we mapped driver positions using Picard LiftoverVcf.

Because DNase I footprint density varies with distance from the transcription start site (TSS) and because models differ in the positional distribution of their identified drivers, we computed a distance-adjusted enrichment rather than a raw overlap fraction. We first empirically estimated the background probability of footprint overlap as a function of distance from the TSS. Specifically, we divided the region surrounding each TSS into 500 bp bins based on absolute distance (1–250 bp away, 251–500 bp away, etc., considering both upstream and downstream positions) and computed the fraction of each bin covered by footprints, averaged across the combined 400 random-split and population-split genes. Let $f_{b}$ denote this fraction for bin *b*. For a given model, let $n_{b}$ denote the number of drivers falling in bin *b*. The expected number of overlaps is then

$$\begin{aligned} \mathbb {E}[\text {overlaps}] = \sum \limits _{b} n_{b} \cdot f_{b}. \end{aligned}$$

Enrichment is then calculated as the ratio of the observed number of overlaps to expected overlaps. To estimate uncertainty, we resampled driver variants with replacement 1000 times, recomputing both observed and expected overlaps for each bootstrap sample to obtain a distribution of enrichment values.

### Linear approximation to Enformer

For each gene *g*, we constructed a linear function $\hat{f}_{g}$ that uses a weighted combination of variant dosages $x_{1}, \ldots , x_{n}$ (with ISM scores as weights) to approximate the predictions of the non-linear Enformer model:

$$\begin{aligned} \hat{f}_{g}(x_{1}, \ldots , x_{n}) = \sum \limits _{i = 1}^{n}\text {ISM}_{i}x_{i}. \end{aligned}$$

This approximation is not exact if the original model contains non-linear dosage effects or interaction terms between variants.

### Rare variant analysis

We assessed the impact of rare variants on the fine-tuned Enformer model’s predictions by replacing minor alleles with major alleles in personal genome sequences during inference. This substitution occurred when allele frequencies—calculated using the training set for each gene—fell below specified thresholds. By applying this procedure across various frequency thresholds, we evaluated the contribution of rare variants to the model’s predictions.

## Supplementary Information

Additional file 1: Supplementary figures and supplementary tables.

## Data Availability

Code to reproduce our analyses and to fine-tune Enformer is available at https://github.com/ni-lab/finetuning-enformer [40]. A snapshot of the version used at the time of writing has been archived on Zenodo: https://doi.org/10.5281/zenodo.19038796 [41]. All fine-tuned models and the processed fine-tuning data are available at https://huggingface.co/anikethjr/finetuning-enformer/tree/main [42]. Our code and fine-tuned models are released under the GNU Affero General Public License v3.0. The RNA-seq data from Geuvadis are available in ArrayExpress under accession E-GEUV-1 [43]. Variant calls from Phase 3 of the 1000 Genomes Project were obtained from http://ftp.1000genomes.ebi.ac.uk/vol1/ftp/release/20130502/. Enformer’s original training data were derived from the data published at https://console.cloud.google.com/storage/browser/basenji_barnyard/data. MPRA data were downloaded from ENCODE using the accession IDs listed in Additional file 1: Table S2.
