# Supplementary material for: Fine-tuning sequence-to-expression models on personal genome and transcriptome data
Source: Genome Biol. 2026 May 25;27:234. doi: 10.1186/s13059-026-04091-1 (PMC13386774; doi:10.1186/s13059-026-04091-1)
Supplement: Supplementary file 1 — Additional file 1: Supplementary figures and supplementary tables. [file 13059_2026_4091_MOESM1_ESM.pdf]

Supplementary Figures

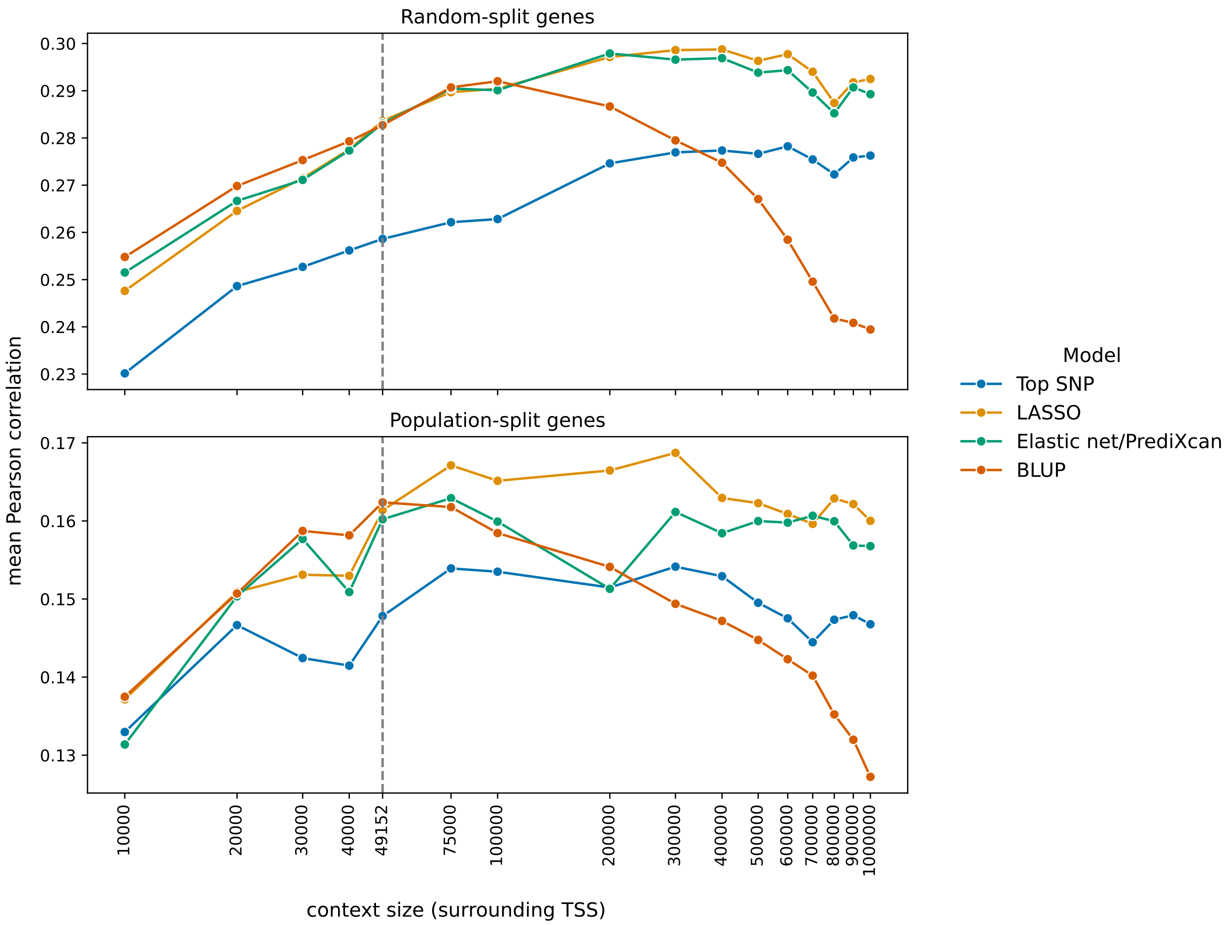

Figure S1: Performance of linear models trained on variants within windows of varying sizes centered on the TSS (a context size of N extends $N/2$ bp on either side). The vertical dashed gray line at $\sim$49.2 kb marks the context size used for training and evaluating all models in all other analyses.

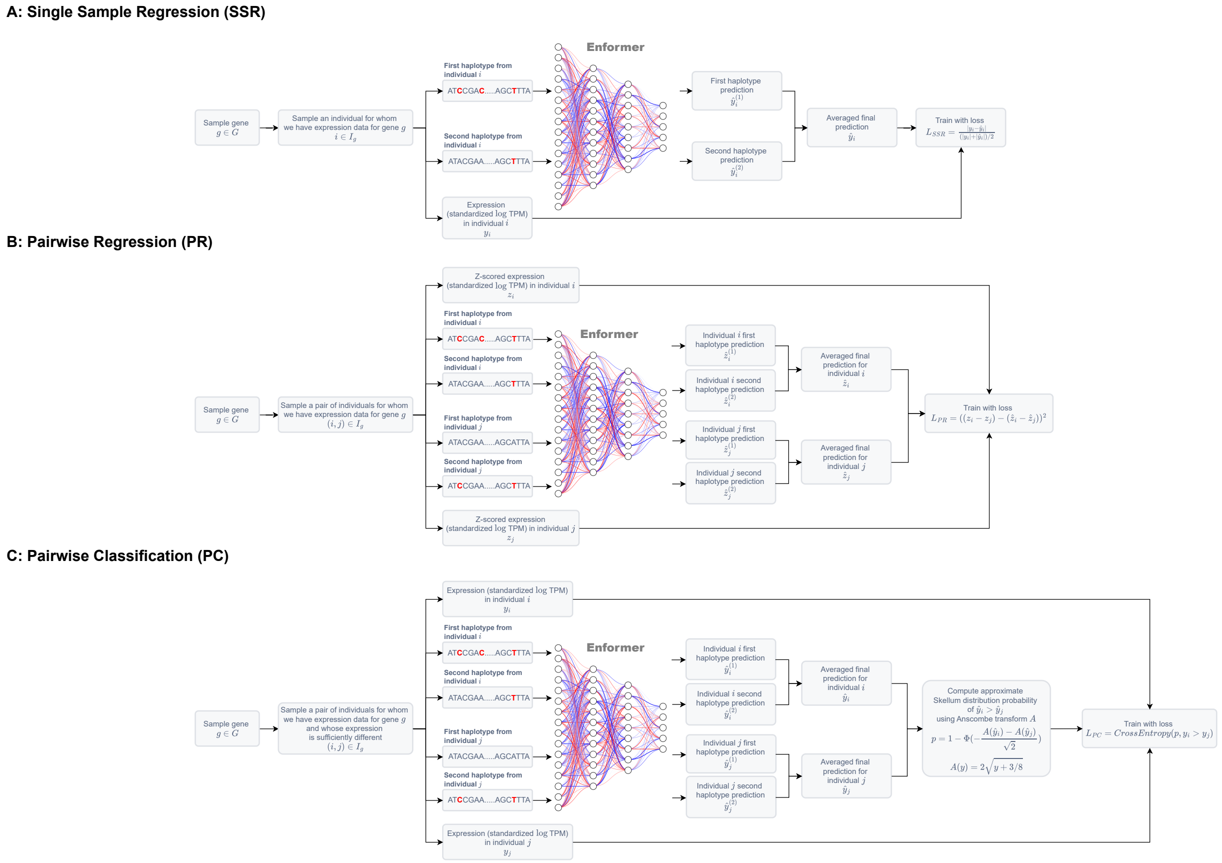

Figure S2: Schematics of the three primary methods used to fine-tune Enformer on paired personal genome and transcriptome data: **(a)** single-sample regression, **(b)** pairwise regression, and **(c)** pairwise classification.

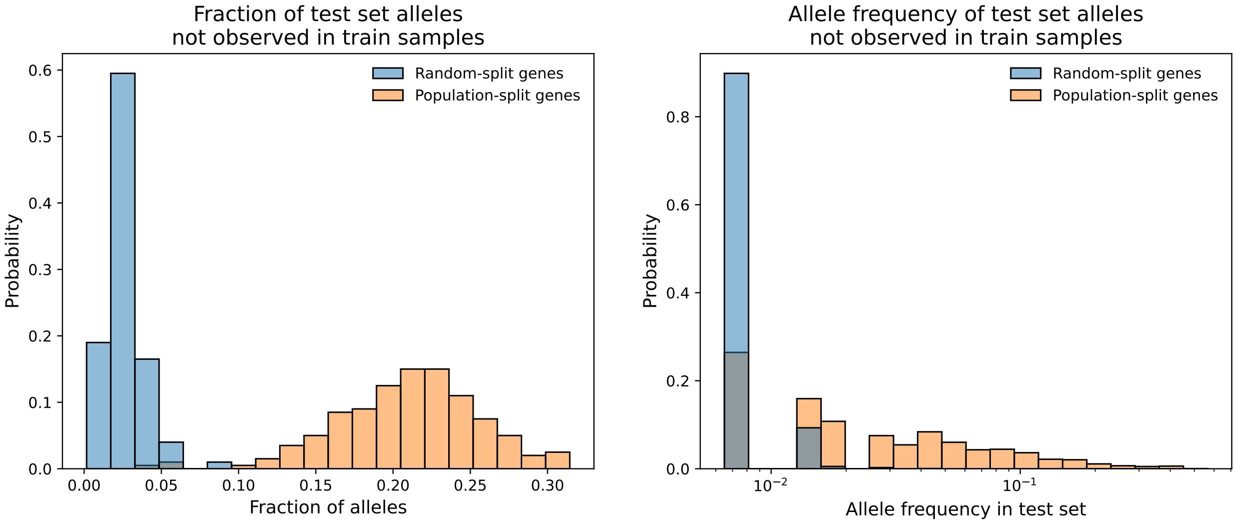

Figure S3: Left: For each gene, we computed the fraction of alleles at polymorphic (non-fixed) positions in the test set that were absent in the training set. We visualize the distribution of these fractions, stratified by gene set. **Right**: For those same alleles that were absent from the training set, we show their allele frequency distribution within the test set, again stratified by gene set.

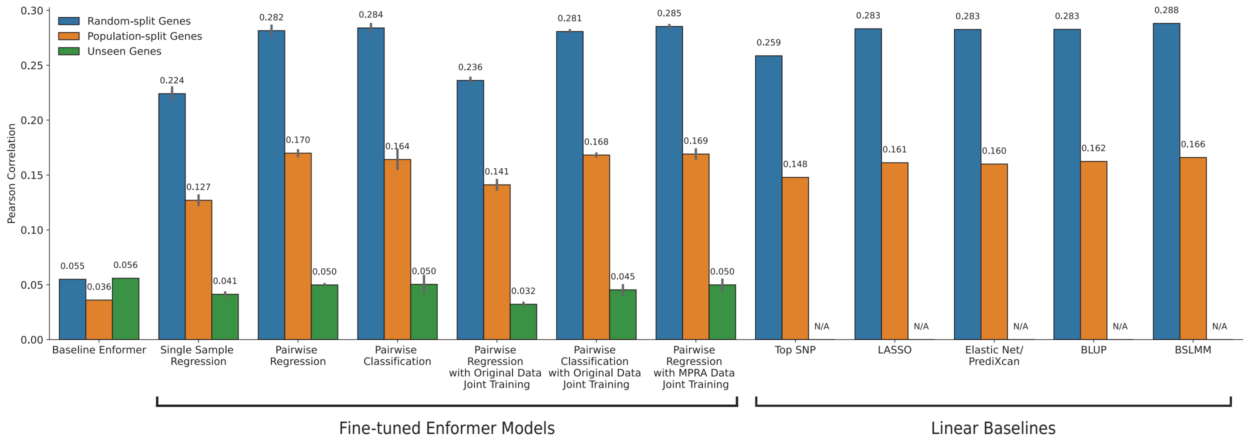

Figure S4: Performance summary of all fine-tuned Enformer models and all baseline methods. Bar heights indicate the mean Pearson correlation across genes. As in Fig. 2b, for the fine-tuned models, this mean correlation is averaged across model replicates, and the error bars show the standard deviation among those replicates.

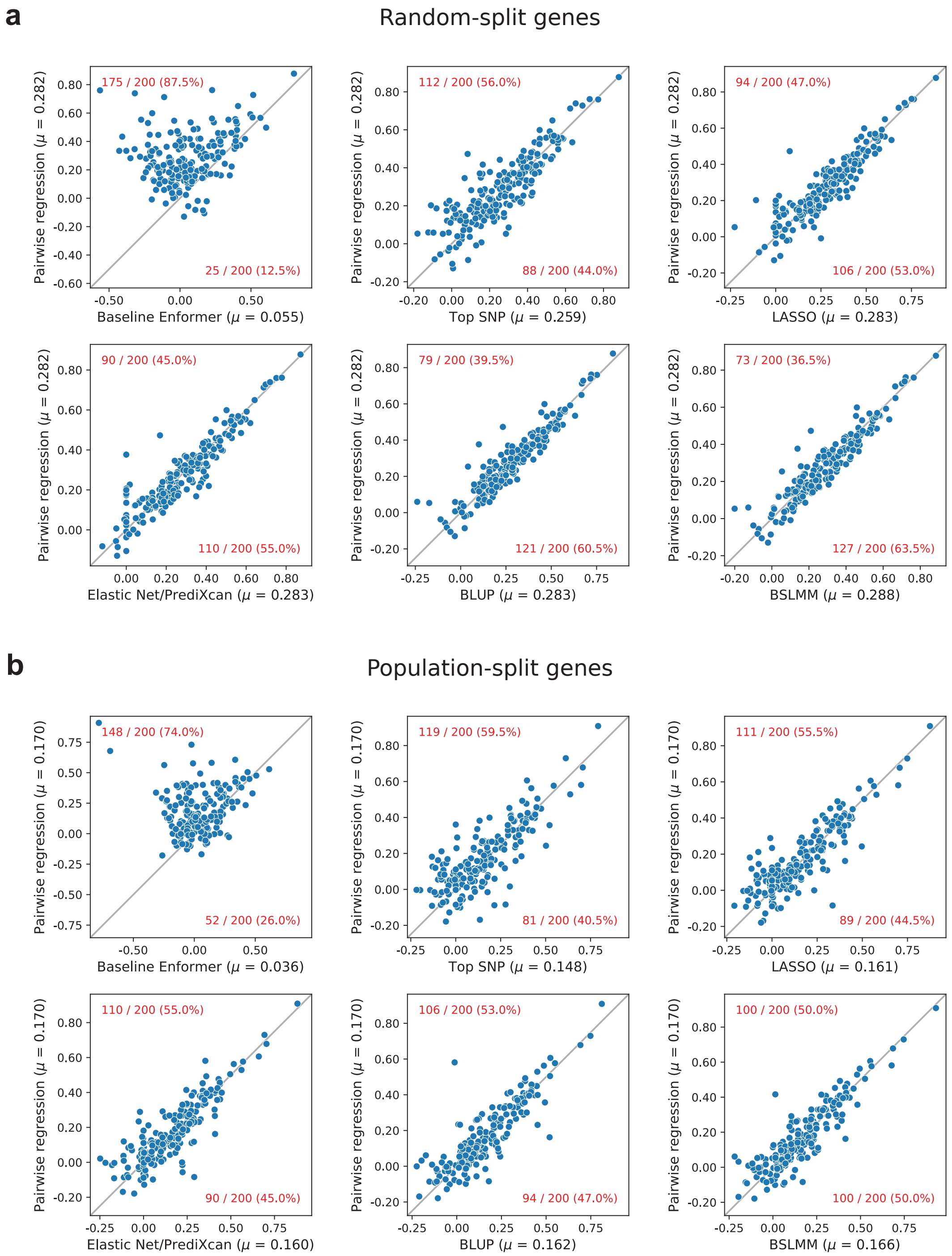

Figure S5: Scatterplots comparing the Pearson correlations of the fine-tuned pairwise regression model with each baseline method on **(a)** random-split genes and **(b)** population-split genes. Each point represents a gene. Red annotations indicate the number and percentage of genes above and below the gray $y = x$ line.

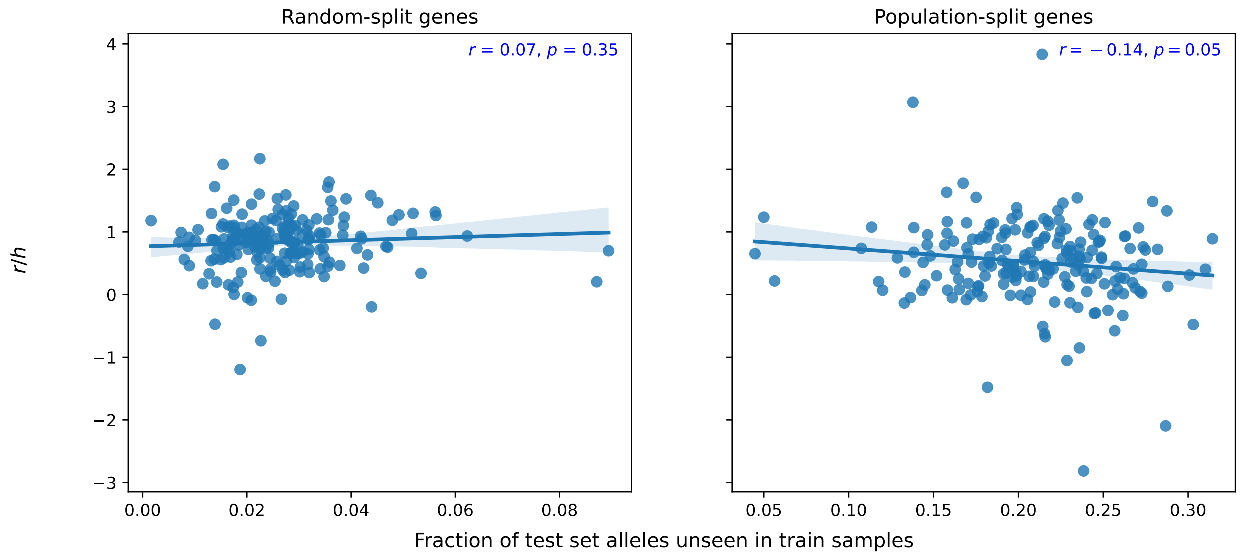

Figure S6: For random-split (left) and population-split genes (right), we plot the fraction of test set alleles that were never seen during training against a normalized performance metric: the model's cross-individual Pearson correlation divided by the square root of the narrow-sense SNP heritability ($r/\sqrt{h^2_{\mathrm{SNP}}}$). A least-squares regression line with a shaded 95% confidence interval is overlaid, and the panel annotations report the Pearson r and its two-sided p-value.

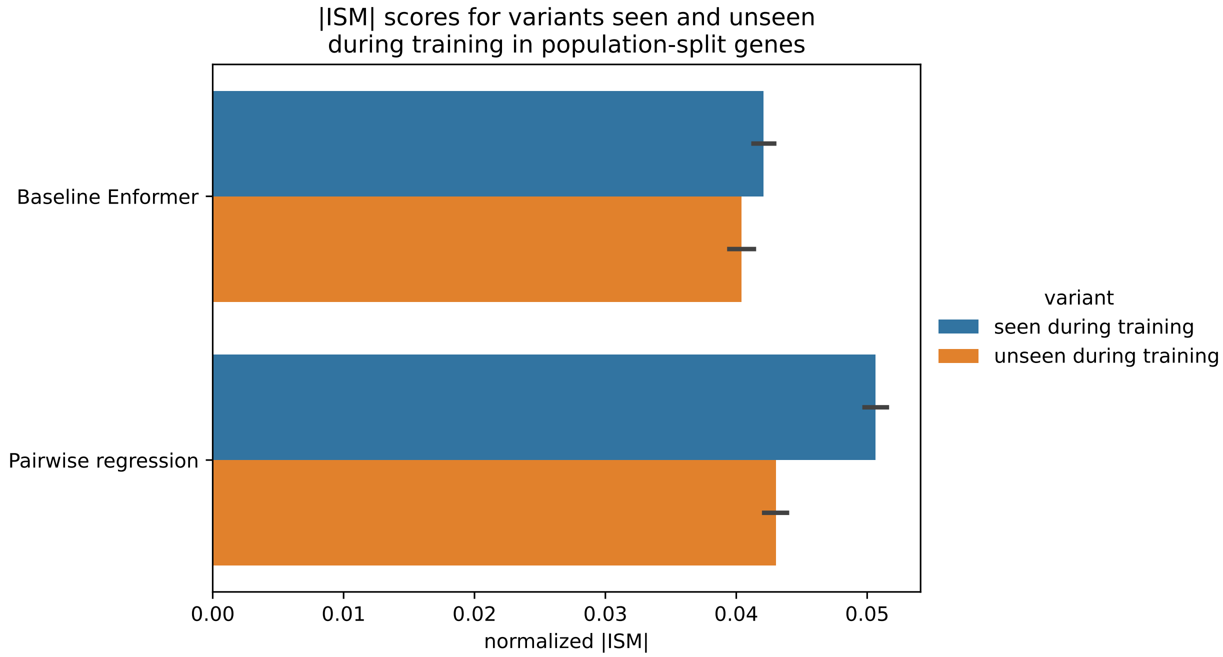

Figure S7: Normalized effect sizes predicted by the baseline Enformer and fine-tuned pairwise regression models for variants in population-split genes. Variants are stratified by whether they were seen or unseen during training of the fine-tuned model (note that all variants are unseen by the baseline model). Normalized |ISM| scores are computed by dividing a variant's absolute ISM score by the maximum absolute ISM score among all variants in our dataset near the same gene. Bar lengths indicate the mean, with error bars depicting $\pm$ 1 standard error.

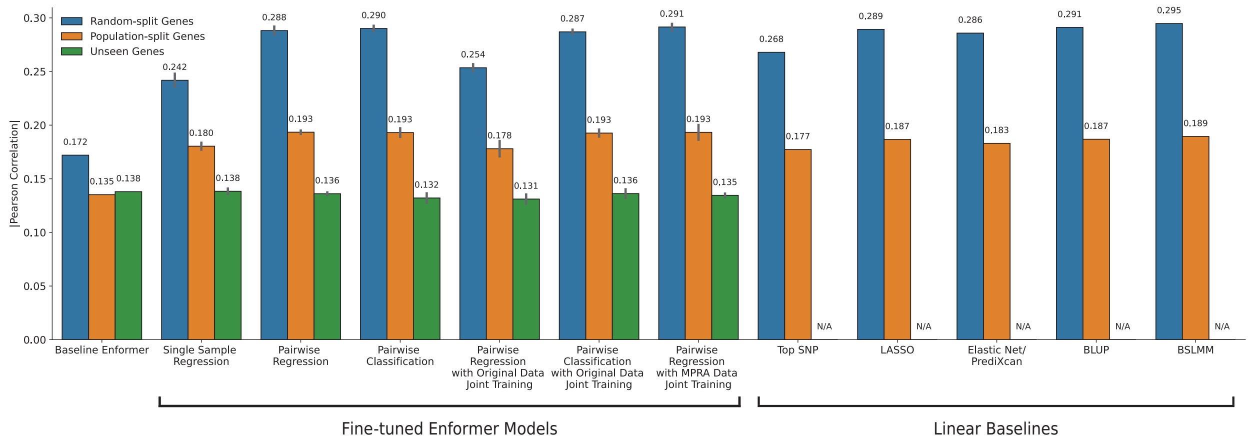

Figure S8: Absolute cross-individual Pearson correlations for fine-tuned Enformer models and baseline methods, with bar heights showing the mean absolute correlation across genes. As in Fig. 2b, for fine-tuned models, this mean was computed separately for each of the three model replicates and then averaged across replicates; error bars indicate the standard deviation across replicates.

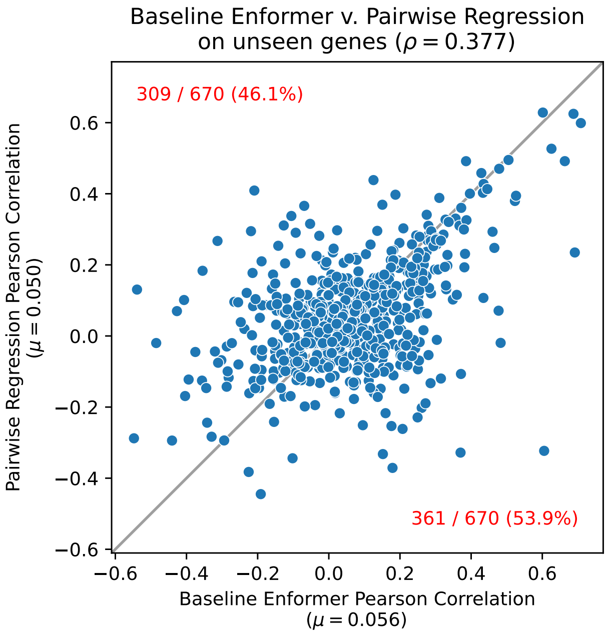

Figure S9: Performance of baseline Enformer and the fine-tuned pairwise regression model on unseen genes. Each point depicts a gene.

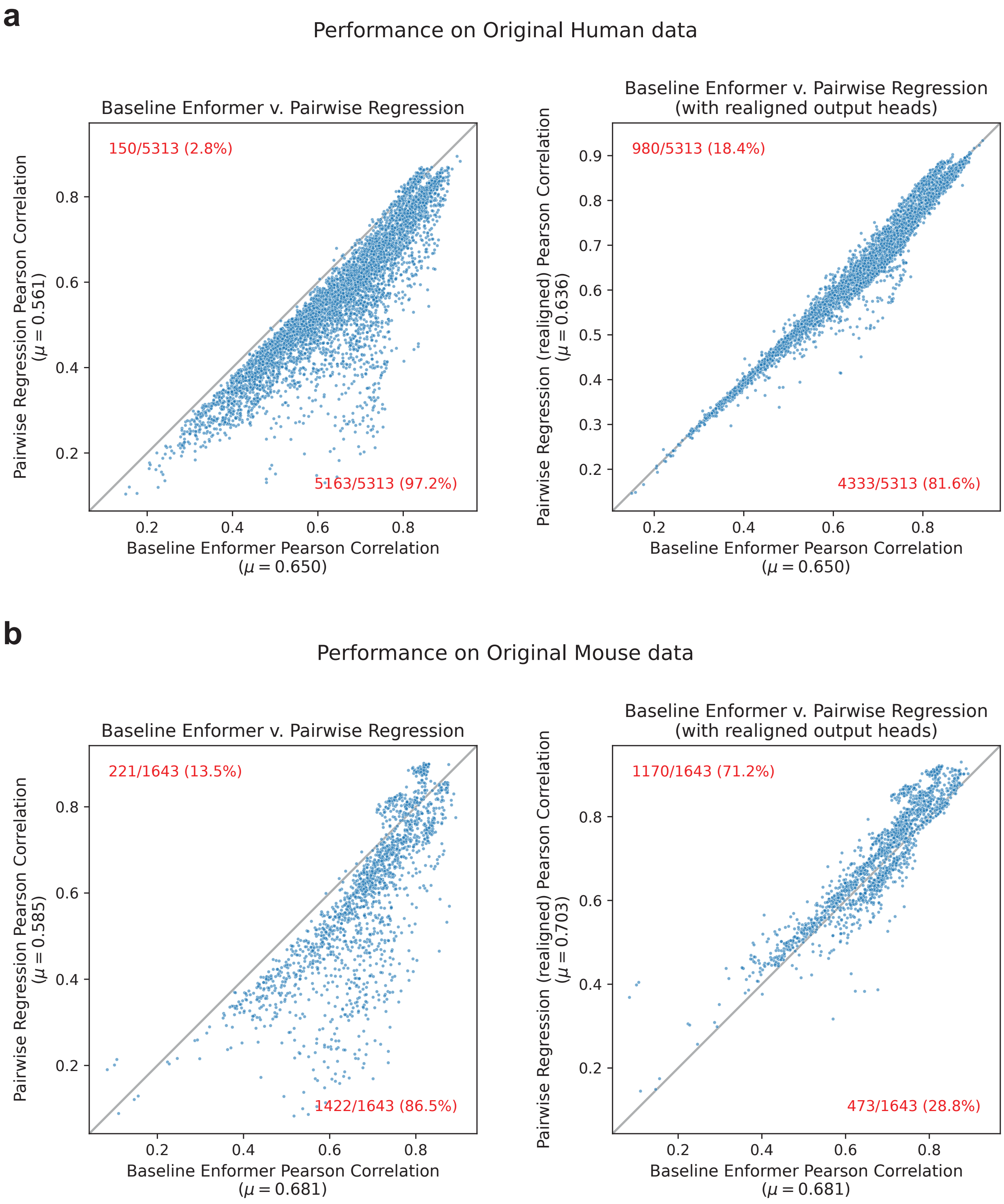

Figure S10: Caption on next page.

Figure S10: Comparison of the performance of baseline Enformer and the fine-tuned pairwise regression model on Enformer's original test set. For each output track, we compute performance as the Pearson correlation between the predicted and observed signal across genomic bins in the test set. Points depict individual tracks, with counts of tracks falling above or below the $y = x$ line indicated. Panel **(a)** shows results for human tracks, while panel **(b)** shows results for mouse tracks. Within each panel, the left scatterplot compares the baseline model to the fine-tuned model as is. Since the personal expression fine-tuning updates the embeddings but not the output heads for the original tracks, these heads may be misaligned. Accordingly, in the right scatterplot, we compare the baseline model to the fine-tuned model after the output heads for the original data are re-aligned by training on the original data, keeping all other parameters fixed (batch size = 32, learning rate $= 1 \times 10^{-4}$, weight decay $= 1 \times 10^{-3}$).

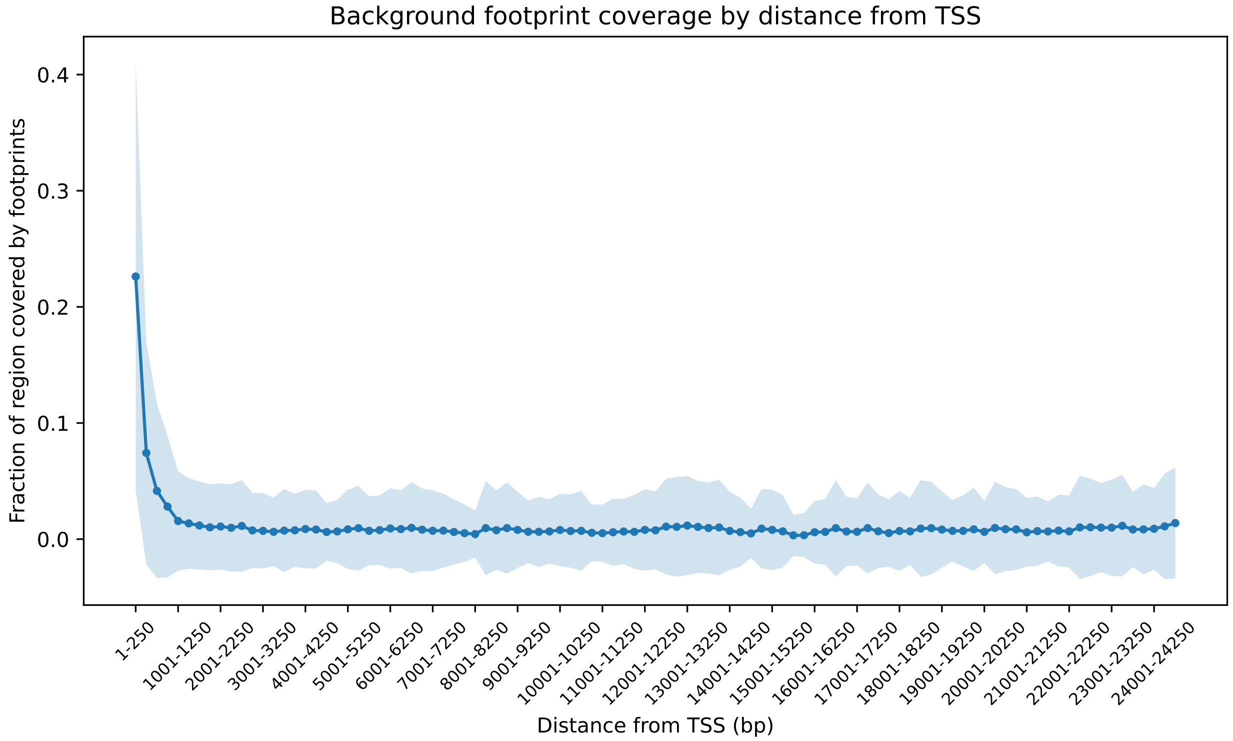

Figure S11: LCL DNase I footprint coverage as a function of distance from the TSS. The solid line shows the mean coverage across 400 random-split and population-split genes. The shaded areas indicate ± 1 standard deviation.

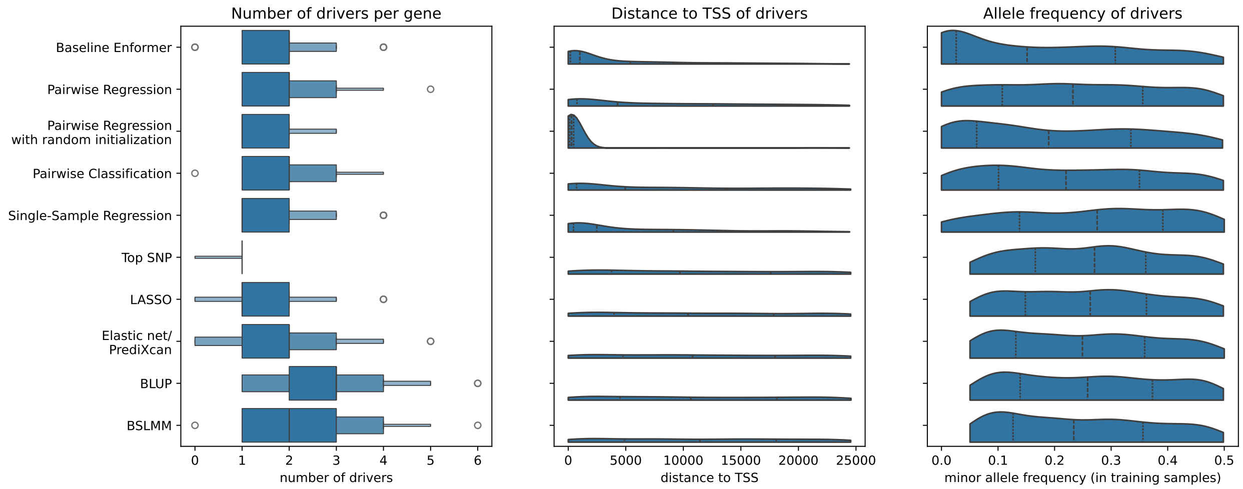

Figure S12: For each model, we identify the variants that most influence predictions, termed "drivers," for both random-split and population-split genes. We analyze (left) the number of drivers per gene, (middle) the unsigned distances of drivers from the transcription start site (TSS) of their corresponding genes, and (right) the minor allele frequencies of drivers, calculated using individuals from the training set. Drivers were identified using the algorithm described in [1]. For fine-tuned models, only a single model replicate was used to identify drivers.

Supplementary Tables

Model type	Training time (hrs.)	Inference time (hrs.)
Pairwise regression	31.1	11.0
Pairwise classification	38.3	11.0
Single-sample regression	40.6	11.0
Pairwise regression (with random initialization)	108.3	11.0
Top SNP	0.4	0.1
LASSO	0.4	0.1
Elastic Net/PrediXcan	0.4	0.1
BLUP	0.4	0.1
BSLMM	32.5	0.1

Table S1: Running time to train models and make predictions on the test set. For fine-tuned models (top block), training was done on a node with 8 NVIDIA A5000 GPUs, 32 CPU cores, and 512 GB RAM, while inference was done on a node with 2 NVIDIA A5000 GPUs, 8 CPU cores, and 128 GB RAM. For linear baselines (bottom block), training and inference were both performed on a node with 48 CPU cores (Intel Xeon Gold 6226, 2.70 GHz) and 376 GB RAM.

Cell type	Number of variants tested	ENCODE accession IDs
GM12878	155283	ENCFF860FTN, ENCFF547AAX, ENCFF394RVK ENCFF382JIM, ENCFF133LIK, ENCFF400HXX ENCFF368WLS
HepG2	357514	ENCFF723IJU, ENCFF590WME, ENCFF660AQG ENCFF409HLO, ENCFF629BDS, ENCFF162EAI ENCFF405HLW, ENCFF810EGX, ENCFF592VHT
K562	357344	ENCFF190GIK, ENCFF484NRP, ENCFF467ZQU ENCFF161KFE, ENCFF793IQS, ENCFF684JVF ENCFF438TIX, ENCFF409FRY
A549	155288	ENCFF244UZB, ENCFF104BLT, ENCFF687ONV ENCFF565QYI, ENCFF514YKU, ENCFF412MJB ENCFF425QDZ
SK-N-SH	357342	ENCFF513ZUR, ENCFF658YTB, ENCFF356VJF ENCFF529TSC, ENCFF153VGD, ENCFF545XTD ENCFF425JFH, ENCFF471LFG, ENCFF796IFY

Table S2: ENCODE accession IDs for the MPRA data published by [2].

References

[1] Sasse, A., Ng, B., Spiro, A.E., Tasaki, S., Bennett, D.A., Gaiteri, C., De Jager, P.L., Chikina, M., Mostafavi, S.: Benchmarking of deep neural networks for predicting personal gene expression from DNA sequence highlights shortcomings. Nature Genetics **55**(12), 2060–2064 (2023)

[2] Siraj, L., Castro, R.I., Dewey, H.B., Kales, S., Butts, J.C., Nguyen, T.T.L., Kanai, M., Berenzy, D., Mouri, K., Wang, Q.S., et al.: Functional dissection of complex trait variants at single-nucleotide resolution. Nature, 1–11 (2026)
